# Supplementary material for: Patterns of Intron Gain and Loss in Fungi
Source: PLoS Biol. 2004 Nov 30;2(12):e422. doi: 10.1371/journal.pbio.0020422 (PMC532390; doi:10.1371/journal.pbio.0020422)
Supplement: Table S1 — Also available at http://genes.mit.edu/NielsenEtAl/. (4.3 MB ZIP). [file pbio.0020422.st001.zip › NielsenEtAl/html/680.html]

AN4778.1.NCU01993.1.MG02633.1.FG08706.1


```
 CLUSTAL W (1.82) Multiple Sequence Alignments - Introns Inserted


Sequence 1: NCU01993.1	436 aa
Sequence 2: MG02633.1	410 aa
Sequence 3: FG08706.1	413 aa
Sequence 4: AN4778.1	412 aa
Alignment Length: 436 aa
Number Identitical Residues: 218 aa
Alignment Score (without introns) 10980


MG02633.1 	MVYVRQEQLPALKEYKYSGVDHSLLSKYVLKPFYTNVVIKIFPMSMA~PNL~ITLTGFTF
NCU01993.1	MVYVRQEKLPNLREYKYSAVDHSLTSKYILKPFYTNVVIKLFPMSMA~PNL~ITLTGFMF
FG08706.1 	MVYVRQKELPALREYKYSGVDHSLLSKYVLKPFYTNFVIKCFPMSMA~PNA~ITLTGFSF
AN4778.1  	MVYIRQHELSNLKNYRYAGVDHSLISRYVLKPFYNNFVIKFFPMSMA2PNA0ITLTGLFF
          	***:**.:*. *::*:*:.***** *:*:*****.*.*** ****** **  *****: *

MG02633.1 	VVANLLTLLWYNPTLDQDCPPWVYYSWALGLFLYQTFDAVDGTQA2RRTHQSGPLGELFD
NCU01993.1	VVGNFLTLLWYNPTLDQDCPPWVYYSWAAGLFLYQTFDAVDGTQA2RRTRQSGPLGELFD
FG08706.1 	VVANFLAMLWYTPSLDQDCPSWVYYSWALGLFLYQTFDAVDGTQA2RRTRQSGPLGELFD
AN4778.1  	VLINLFTVLYYNPSLDQDCPPWVYASCAIGLFLYQTFDAVDGIQA2RRTKQSGPLGELFD
          	*: *::::*:*.*:******.*** * * ************* ** ***:**********

MG02633.1 	HG~VDAVNTSLEVLLFAGSQNMGQSWYTVAVLFSS1LLTFYVQTWDEYHTKTLTLGIVNG
NCU01993.1	HG~VDALNTSLECLIFAASQNMGQSWYTVATVFAS~LLTFYVQTWDEYHTKTLTLGIVNG
FG08706.1 	HG~VDACNTSLEVLLFAASQNMGQSWQTVAVLFAS1LLTFYVQTWETYHTKTLTLGIVNG
AN4778.1  	HS1VDACNTALGVLIFAGVMNLGQTWATVLTLFGS1TMTFYVQTWDMYYTQVLTLGIVSG
          	*. *** **:*  *:**.  *:**:* ** .:*.*  :*******: *:*:.******.*

MG02633.1 	PVEGVLTIVVVFILTGYLGGASFWQQPMLPTLGVP-VFEF------LPSFLYKLSFTQIW
NCU01993.1	PVEGILILVGVYTLTGYLGGASFWQQGMLSTLGVPQTLTFGTTALTLPSFLYNFSFTEWY
FG08706.1 	PVEGVLAIVLVFIFTGYVGGAHFWQQSMFQTIGVPSTVGV-------PSFIYNLTFTEWY
AN4778.1  	PVEGVLTLCVVFGFTAYMGGGSFWHQPMFETIGVP-KLEF------IPKQLYDLPFTQWY
          	****:* :  *: :*.*:**. **:* *: *:***  . .       *. :*.:.**: :

MG02633.1 	MVQGTLVLVHNTLESCRNVIKARRAQGDRSRYALVGLLPFFGTWSCILAYLFLQPNIRQG
NCU01993.1	MVQGTVVLVYNTVESARNVIRARRARGDRSRYALVGLLPFFATWALVVAYLFLQPNILHG
FG08706.1 	LVQGSIMLVFNTVESSLNVIRARRARGDRSRGALLGLVPFFSIWSMVVAYLYLQPKIRQC
AN4778.1  	LIYGAVMLFFATGSSIAHVIQVRKERGKDSIGPLFGILPLALTWVVVPAYLYLNPTILEN
          	:: *:::*.. * .*  :**:.*: :*. *  .*.*::*:   *  : ***:*:*.* . 

MG02633.1 	HLVPFALFAGIVNAYSVGQIITAHLVHLKFPYTNVLTLPLAWGVVDSLGPWLLSTFG---
NCU01993.1	HLVPFVLFAGIVNAYSVGKMITAHLVKLDFPYWNVMVIPLGLGVFDSLGPVIHKYAPDGL
FG08706.1 	HLVPFALFAGLVNAYSVGQMITAHLVHLRFPYWNVLGLPLAFGVIDSLGPIFQQHLG---
AN4778.1  	YLVPFALYVGLVNAYAVGRIICAHLVHQDFPYFNILLGPLALAVVDSAGALFG-------
          	:****.*:.*:****:**::* ****:  *** *::  **. .*.** *. :        

MG02633.1 	--FGWPSALGDGVYQVAFMFCLLGMAIGVYGSFV~VDVIVTICDYLDIWCLTIKHPYVEG
NCU01993.1	KNLGWPSALGDGVYQVAYMFCMLGMAVGVYGSFV~VDVIVTICDYLDIWCLTIKYPYVEG
FG08706.1 	--VGWPSALGDSVYQVAFMFMMLGTAVGVYGSFV~VDVIVTICDYLDIWCLTIKHPYVEN
AN4778.1  	---VWSSTLIGTIGQPAFVFLCLGLGLGVYGSFV0HDIITTICDYIDIWCLTIKHPYVPE
          	    *.*:* . : * *::*  ** .:*******  *:*.*****:********:***  

MG02633.1 	QDNGKKTN--------------
NCU01993.1	QDDPKVSKQVVGSKGELKEKRG
FG08706.1 	EGPQANGTKKD-----------
AN4778.1  	ESVNGNVVRAAKKNL-------
          	:.          ..
```
